# Determination of Saturation Conditions of the Aluminum Metal Matrix Composites Reinforced with Al_2_O_3_ Sinter

**DOI:** 10.3390/ma16186106

**Published:** 2023-09-07

**Authors:** Paweł Szymański, Paweł Popielarski, Dorota Czarnecka-Komorowska, Robert Sika, Katarzyna Gawdzińska

**Affiliations:** 1 Institute of Materials Technology, Poznan University of Technology, Piotrowo 3 Str., 61-138 Poznan, Poland; pawel.szymanski@put.poznan.pl (P.S.); pawel.popielarski@put.poznan.pl (P.P.); dorota.czarnecka-komorowska@put.poznan.pl (D.C.-K.); 2 Faculty of Marine Engineering, Maritime University of Szczecin, Willowa 2-4, 71-650 Szczecin, Poland; k.gawdzinska@pm.szczecin.pl

**Keywords:** casting, aluminum metal matrix composites, castability, energy balance

## Abstract

Aluminum metal matrix composites (Al MMCs) are a class of materials characterized by being light in weight and high hardness. Due to these properties, Al MMCs have various applications in the automobile, aeronautical and marine industries. Ceramic-reinforced Al MMCs in the form of sinters are known for having excellent abrasive properties, which makes them an attractive material in certain fields of technology. The biggest problem in their production process is their low ability to infiltrate ceramics with alloys and consequently the difficulty of filling a ceramic preform. The castability of such composites has not yet been researched in detail. The aim of this study was to create aluminum metal matrix composite castings based on aluminum alloys (AlSi11) reinforced with an Al_2_O_3_ sinter preform using a Castability Trials spiral mold, and then to determine the degree of saturation with the liquid metal of the produced ceramic shaped body (Castability Trials spiral). For the selected AlSi11 alloy, the liquidus (Tl) and solidus (Ts) temperatures were determined by performing thermal-derivation analysis during cooling, which is Tl—579.3 °C and Ts—573.9 °C. The resultant pressure necessary for the infiltration process was estimated for the reinforcement capillaries with the following dimensions: 10, 15, 20, 25, 30 and 35 microns. The following values were used to determine the capillary pressure (Pk): surface tension of the alloy—σ = 840 mN/m; the extreme wetting angle of the reinforcement by the metal—θ = 136°. It has been experimentally confirmed that for the vacuum saturation process, the estimated resultant pressure enables saturation of reinforcement with capillaries larger than 25 microns, provided that the alloy temperature does not drop lower than the infiltration temperature. After the experiment, the time and route of the liquid metal flow in the spiral were determined. On the basis of the obtained values, a simulation was developed and initial assumptions such as saturation time, alloy temperature, reinforcement and mold temperature were verified. The energy balance showed that the saturation limit temperature was Tk = 580.7 °C for the reinforcement temperature of 575 °C. In contrast to the above, the assumption that the temperature of the metal after equalizing the temperature of the composite components must be higher than the liquidus temperature (Tliq = 579.3 °C) for the aluminum alloy used must be fulfilled. After the experiment, the time and path of the liquid metal flow in the spiral were determined. Then, on the basis of the obtained values, a simulation was developed, and the initial assumptions (saturation time and temperature) were verified.

## 1. Introduction

The casting properties of an aluminum alloy have a significant impact on the mold-filling process, and they have an influence on the structure and properties of the casting, especially of the composite casting, where a reinforcing phase appears. One of the most important of these features is the castability, understood not only as the distance made by the alloy of the composite matrix from the moment of pouring to complete infiltration of the reinforcing structure and solidification, but also as a set of features characterizing the flow of liquid metal in the mold cavity, i.e., the velocity and time of a flow, the level of reinforcement infiltration with a liquid metal, or changes in the temperature of a solidifying metal [1,2,3,4,5,6].

The knowledge of temperature changes or the velocity of filling the mold cavity with liquid metal enables, at the later stage, modelling of the process of reinforcement infiltration with a liquid alloy and filling the mold cavity [5]. Determining the castability, especially in the case of the reinforcing phase in composites (due to the complex nature), is exceedingly difficult by analytical means. It depends, as already mentioned, on many variable factors that are related to the properties of the liquid alloy (the chemical composition, viscosity, surface tension, and content of oxides and gases), mold properties (the specific heat and thermal conductivity of the mold material and surface roughness), and pouring conditions (the metal temperature, mold temperature, gating system height, pressure, and rate of filling).

These factors affect the quality of the casting [7]. The formulas [8,9] given in the literature are approximate. Therefore, the castability of casting alloys for a given group of castings (especially composite ones) is determined experimentally, or for comparative purposes, in special casting tests. Marisa Di Sabatino et al. described, in their work [10], the effect of the aluminum alloy composition on the fluidity, macrosegregation, and defects resulting from the casting process. They also discussed predictive models for castability prediction and data on other process parameters. Ray S. focused on the problems of the quality of composites with particles and a metal matrix and their relationship with the process variables and characteristics of a given casting process [11].

Modelling in a foundry enables the design of new materials and technologies [12,13]. According to the BN-80/4051-17 standard [14], there is a spiral test in which the castability is determined by the length of the spiral cast in a standardized manner. Attempts to test the castability of a composite with an Al matrix and reinforcement in the form of SiC particles using the castability spiral were presented by Sourav Kayal et al. In article [15] the authors presented the results of the influence of the pouring temperature (Tp) and at different weight fractions of SiC on the castability of aluminum silicon metal matrix composites in thin-walled castings with wall thicknesses of 3, 4, and 5 mm, respectively.

The analysis was performed on an aluminum silicon alloy that was reinforced with 5 wt.%, 10 wt.%, and 15 wt.% of SiC with an average 400 mesh size and a pouring temperature varied from 680 °C to 725 °C. Statistical analysis showed that pouring temperature had a much higher influence on the castability in comparison with the influence of the reinforcing particle [15]. In the case of infiltrated composites, this is a difficult process due to the “stiffness” of the reinforcing phase that gives the proper shape of the product. The process of producing infiltrated composites has been described in detail in the literature [16,17,18,19,20,21]. Calin R et al. determined the effect of powder size on the infiltration efficiency related to the fluidity of an Al alloy in the production of MgO-reinforced composites using the vacuum infiltration method [22]. In this work and from the author’s experience, in the case of composites with saturated reinforcement, we can see clearly that the infiltration process depends on the castability of the matrix and should be carried out based on the rules of infiltrating the reinforcement with a liquid matrix. During the infiltration of the reinforcement with metal, partial processes may occur, which may be advantageous from the point of view of filling the capillary spaces of the reinforcing structure, the composite production, or undesirable, such as the formation of defects (insufficient saturation). These include, among other things, not filling the reinforcement and the formation of gas occlusions.

During infiltration, in addition to overcoming the initiation pressure and the flow of an alloy into the spaces (channels) with the most favorable flow conditions (these places are located in areas with a locally increased preform porosity) and filling the reinforcement areas with increased hydraulic resistance, the decisive stage takes place, i.e., filling capillary spaces, formed by particles in contact or located close to each other [23].

**Figure 1:**
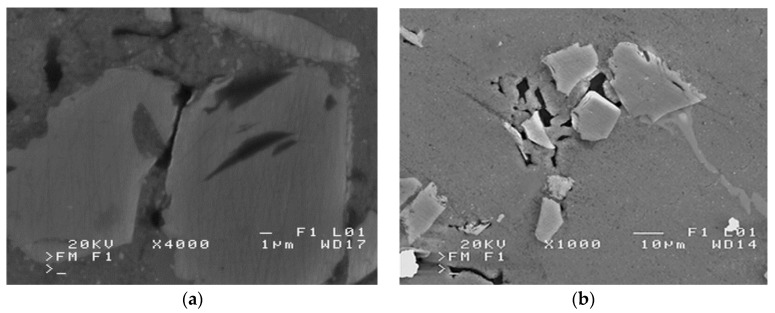
Scanning electron microscope (SEM) micrographs of incomplete filling in the infiltrated SiC/silumin composite of (own research): (**a**) reinforcement space, and (**b**) capillary space between reinforcement elements.

The phenomenon of incomplete filling of capillary spaces, occurring in infiltrated composite castings is illustrated in Figure 1. The capillary pressure value (*p*_kap_) for this case can be determined on the basis of the Young–Laplace equation [16]:

(1) $$p_{\mathrm{kap}}=\frac{4\sigma }{d_{min}}\cos\theta$$

where

*σ*—the value of the liquid surface tension [N/m],
*d_min_*—substitute diameter [m], and
*θ*—the contact angle [°].

**Figure 2:**
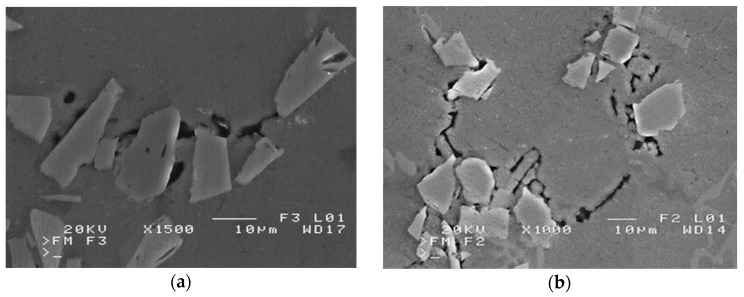
SEM micrographs of defects in a SiC/silumin composite (own research): (**a**) clearly visible slight blisters in the composite structure between the matrix and the reinforcement particles, and (**b**) discontinuities of the matrix structure.

Therefore, in the case of unfavorable wetting of the reinforcing phase surface with liquid metal, in capillaries formed in preforms (made of disordered compressed particles), where there are numerous areas of mutual contact of sinter particles, we are dealing with spaces whose complete filling with a liquid alloy requires high pressure values, theoretically going to infinity. Achieving complete filling of intermolecular spaces is therefore impossible, and each composite infiltrated with liquid metal with a reinforcement made of disordered particles, also in the form of a sinter, is porous [16]. These can be both the porosities of the composite structure (Figure 2a) or discontinuities of the matrix structure (Figure 2b), as illustrated in Figure 2.

**Figure 3:**
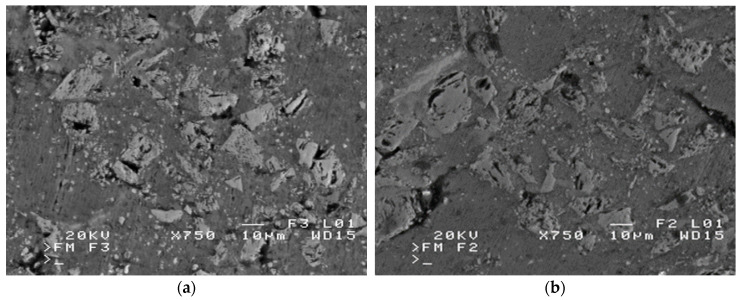
SEM micrographs of the defects of a SiC/silumin composite reinforcement (own research): (**a**) brittle breaks of particles, and (**b**) chipping of reinforcement elements.

The use of a pressure higher than necessary for the required (incomplete) filling of the capillaries is undesirable as it may lead to deformation or displacement of the ceramic preform. Disproportionate pressure also introduces excessive stresses in the particles in the areas of the liquid metal front, which can lead to cracks, chippings, tears, and breaks in the reinforcing structure as shown in Figure 3.

Incomplete filling of the ceramic preform capillaries may occur during the infiltration of the preform with liquid alloy as a result of the solidification of the matrix metal [23]. To avoid this, after the temperature of the composite components is even, the temperature of the alloy must be higher than the temperature of the liquidus metal matrix. This condition can be fulfilled by selecting the initial temperature of the matrix and ceramic preform. They can be calculated from the thermal balance [16,24,25,26] of the composite casting production process, which will be presented later in this paper.

There are no studies related to the production of composite castings with saturated ceramic reinforcement using the melted/lost model method. Composite materials belong to the group of difficult-to-machine materials, so it becomes advisable to produce shape castings while minimizing their machining, which is associated with the economic aspect. The use of 3D printing in the processes of manufacturing the reinforcing structure for MMC enables obtaining any shape of composite castings. The tests presented in this work are intended to present the indicated technology, characterize the process parameters and assess the degree of saturation of the reinforcing structure, which enables assessing the quality of composite castings.

The aim of this work was to produce composite castings based on aluminum alloy (AlSi11) reinforced with Al_2_O_3_ sinter. The parameters of the process of sinter infiltration with a liquid matrix metal were determined along with the energy balance of the composite casting manufacturing process. The ceramic preform was in the shape of a spiral that was placed in the mold. The ceramic mold was made using the lost-wax casting process [27,28,29,30], and the spiral, on the basis of which the degree of infiltration was assessed, was produced by the rapid prototyping fused deposition modelling (FDM) technique with high-impact polystyrene (HIPS) [31,32,33].

Three-dimensional printing based on modelling (FDM) with a thermoplastic polymer can allow for the integration and embedding of objects during 3D printing, and FDM-based 3D printed elements typically do not require any final processing and finishing, which was confirmed by Yuen [34]. There is a wide range of filaments used in the additive technique [34,35]. The choice of the type of filament is primarily determined by the maximum temperature to which the 3D printer head can heat up [34,35,36]. The FDM technology makes use of polymers such as polylactide (PLA), high-impact polystyrene (HIPS), and acrylonitrile-butadiene-styrene copolymer (ABS), poly(ethylene terephthalate) copolymer with ethylene glycol (PET-G), and polyamide 6 (PA6), which are used due to the economics and shorter printing process.

HIPS is a modification of polystyrene (PS) resulting from the addition of butadiene rubber [37]. This material has a high impact strength, is easily thermoformed, and does not requiring pre-drying. Due to this, it is possible to construct even complex spatial forms. However, the use of rubber reduces the tear strength, the transparency of the material, the hardness, and the stability of the form at high temperatures. HIPS is recommended for internal applications [37,38,39].

In this study, HIPS was used due to the ease of creating layers, the satisfactory durability of the material, appropriate stiffness and flexibility, and the low price of this material. During pouring a mold with a liquid metal alloy with a melting point higher than that of polystyrene, at approximately 240 °C, a complete burn of the polystyrene appears, without a large amount of residual model material, which significantly affects the quality of the casting surface. The instrumentation made with the FDM method from high-impact polystyrene (HIPS) enables its complete gasification in the furnace without ash formation [17].

This material is widely used, although the disadvantage of HIPS is that when heated, it produces a slightly unpleasant styrene smell, and its vapors contain harmful substances. Therefore, when printing, proper care and room ventilation are required to remove these acrid vapors.

The use of the FDM technique while creating molds may be beneficial from the point of view of creating the final shape of the product in good quality [40]. The parameters of the process of sinter infiltration with liquid matrix metal were determined along with the energy balance of the composite casting manufacturing process. The experimental results were used for the numerical simulations of the casting process to predict the temperature distribution in the mold and preform.

The experiment aimed at determining the degree of saturation of the produced ceramic body with liquid matrix metal and the determination of the basic parameters of the impregnation processes was carried out in the following stages:

– Creating a 3D model of a fitting in the form of a spiral with HIPS.
– Ceramic coating of printed HIPS fittings.
– Firing ceramic shapes (creating a spiral-shaped reinforcing structure–ceramic sinter).
– Composite casting, consisting of preparing the mold, placing reinforcement in it, pouring liquid metal into the mold, and solidifying, cooling, and removing the casting from the mold. The casting operation was performed eight times with different process parameters.
– Assessment of the degree of saturation of the fitting by measuring the distance from the face of the spiral using microscopic methods.
– Performance of the energy balance of the composite casting manufacturing process.
– Verification of the casting process by the finite difference method (FDM).

## 2. Methods

### 2.1. Optical Microscopy

To conduct the evaluation of the reinforcing preform infiltration, optical microscopy was used. The surface morphology of the samples was taken using an optical polarized light microscope (Nikon Eclipse MA200, Kanagawa, Japan), equipped with the Nikon Imaging Software v.4.50 (NIS)—Elements Basic Research (BR) (Praha, Czech Republic). Optical microscopy (OM) enables assessing the filling of the reinforcing structure with liquid metal, by indicates porosity and other defects of the composite microstructure. Using OM, it is possible to assess the size of a filled or unfilled capillary in the aluminum matrix reinforcement structure with liquid metal. Observations were made on properly prepared microsection specimens. The microsections were not consumed. The enlargements were selected individually to assess the microstructure of the composites.

### 2.2. Energy Balance of the Process of Producing Composite Castings

The energy balance of the composite casting production process was conducted with the use of empirical data, which were obtained from numerical simulations. The 3D model of the cast-mold system was prepared in CAD Autodesk Inventor and exported in STL format to the simulation code. Simulation tests were per-formed using NavaFlow&Solid 6.6 release 1 simulation code (NovaCast Systems AB, Ronneby, Sweden). A mesh was made using the Finite Volume Method (FVM) with a size of 403,440 elements, of which 27,848 mesh elements were cast. A quasi-equilibrium model without segregation was used. Boundary conditions and thermophysical data of the materials were determined on the basis of validation studies of the thermal model. In the experiments, the temperature distributions were carried out using contact methods (type K thermocouples) and non-contact methods [41] using the Flir E6XT (Wilsonville, OR, USA) and Vigo V50 thermal imaging cameras (Ożarów Mazowiecki, Poland) and the Raytek two-color pyrometer (Santa Cruz, CA, USA). An energy balance shows the flow of energy for a given technological process. Within its framework, the energy demand of the process and its effect are compared. In this work, the influence of the reinforcement and the matrix on the formation of the casting structure was used, including such factors as: the specific heat of the reinforcement and the aluminum matrix, the influence of the temperature of the components as a function of the volume and density of the casting. Empirical data were used for the simulation relationship of creating composite shape castings using the lost-wax casting method. To perform the energy balance of the composite casting production process, we used the dependency [16,23,24,25,26]:

(2) $$V\cdot P\cdot c_{\mathrm{met}}\cdot \rho_{\mathrm{met}}\cdot \Delta T_{\mathrm{met}}=V\left(1-P\right)c_{\mathrm{zbr}}\cdot \rho_{\mathrm{zbr}}\cdot \Delta T_{\mathrm{zbr}}$$

where

*V*—the composite volume,
*P*—the porosity of the ceramic preform,
*c*_met_—the specific heat of infiltrating metal matrix,
*c*_zbr_—the specific heat of the reinforcement material,
*ρ*_met_—the density of the infiltrating metal matrix,
*ρ*_zbr_—the reinforcement density,
Δ*T*_met_—the temperature decrease in the liquid matrix, and
Δ*T*_zbr_—the temperature increase in the reinforcement material.

As,

(3) $$\Delta T_{\mathrm{met}}=T_{\mathrm{met}}-T_{k}$$

(4) $$\Delta T_{\mathrm{zbr}}=T_{k}-T_{\mathrm{zbr}}$$

Therefore,

(5) $$T_{\mathrm{met}}-\Delta T_{\mathrm{met}}=T_{\mathrm{zbr}}+\Delta T_{\mathrm{zbr}}$$

where

*T*_met_—the initial temperature of the liquid matrix,
*T*_zbr_—the initial temperature of the reinforcement, and
*T_k_*—the temperature of the infiltrated composite.

## 3. Materials and Samples Preparation

The tooling for the production of reinforcement was printed on a Zortrax M200 printer (Zortrax S.A, Olsztyn, Poland) made of high-impact polystyrene (HIPS) in a natural color with the trade name Filament Devil 1.75 (Devil Design Ryszka Mateja Sp. J., Mikołów, Poland) with a density of 1.06 g/cm^3^ and a diameter of 1.75 mm [42].

**Figure 4:**
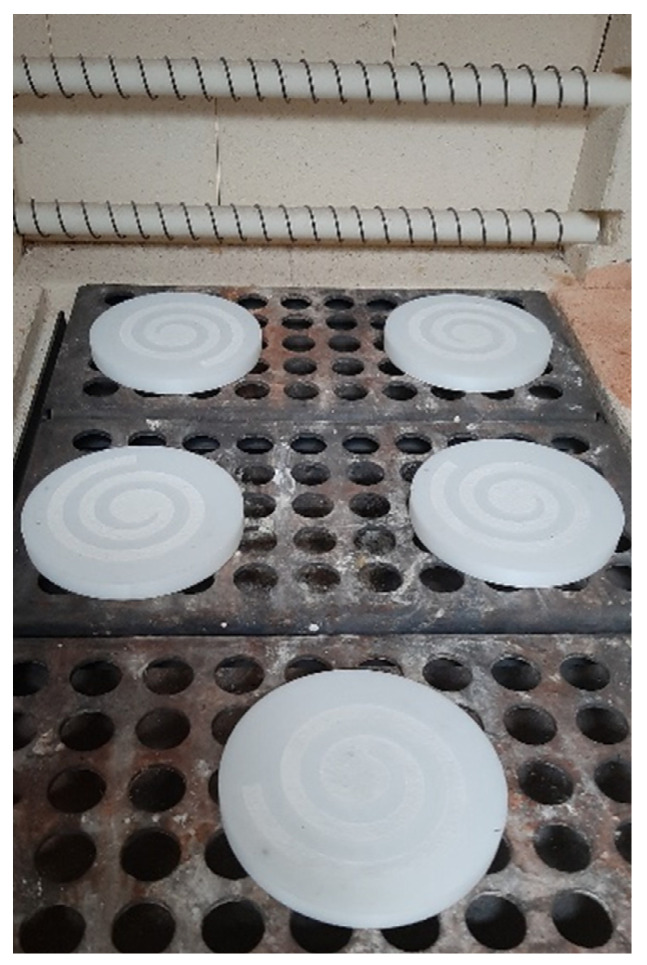
Macroscopic picture of the instrumentation for the production of reinforcement (HIPS spiral with ceramic mass).

The printed fittings (spirals) were covered with a ceramic mass, which was a mixture of aluminum oxide powder (Al_2_O_3_) with a grain size in the range of 100 µm and sodium silicate with a mass fraction of 4%. This mass was initially hardened by blowing with CO_2_ gas and finally placed in the furnace. Annealing was carried out in two stages. In the first stage, the fittings involves the firing of polystyrene with a mass of Al_2_O_3_ in a mold firing furnace. This type of furnace is equipped with a heated drain for molten polymers, special filters and ventilation, with the ceramic mass were burned out at the temperature of 520 °C. Figure 4 shows the fittings before the burning process. During the first stage of burning, the HIPS fitting was completely gasified.

The second stage consisted of a 22 h annealing cycle in a high-temperature ceramic furnace at 1500 °C. During this cycle, the ceramic and water glass particles were partially fused. Selected properties of the obtained sinter are presented in Table 1 [43].

**Figure 5:**
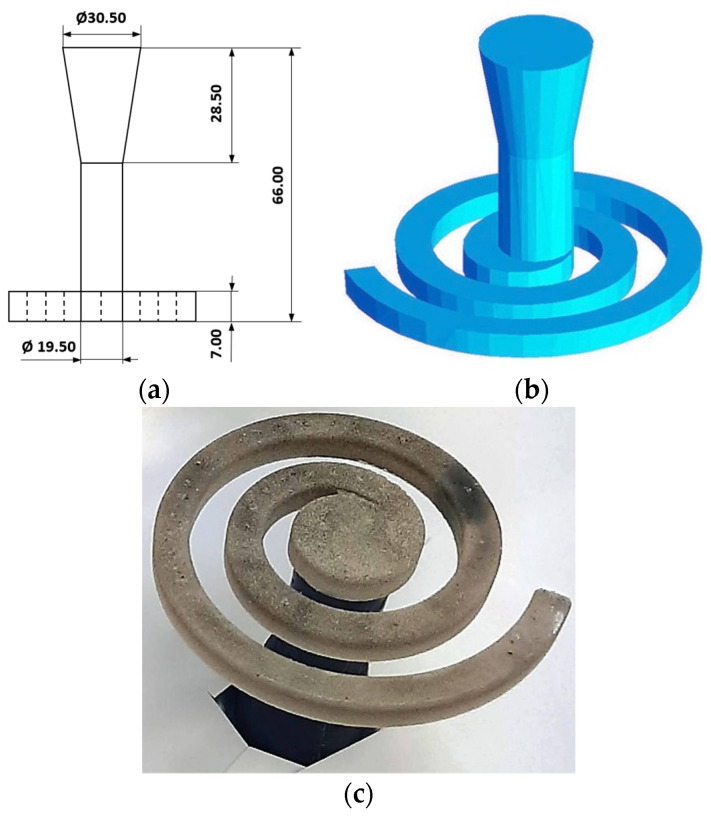
Ceramic spiral: (**a**) Technical drawing of the 2D model, (**b**) 3D model system, and (**c**) view of a paraffin-impregnated ceramic spiral with a wax gating system.

Obtained with aluminosilicates, the spiral-shaped ceramic sinters were impregnated with paraffin. This impregnation aimed to protect the fitting against penetration of the liquid molding mass. A wax gating system was mounted to the fitting impregnated with paraffin as shown in Figure 5.

**Figure 6:**
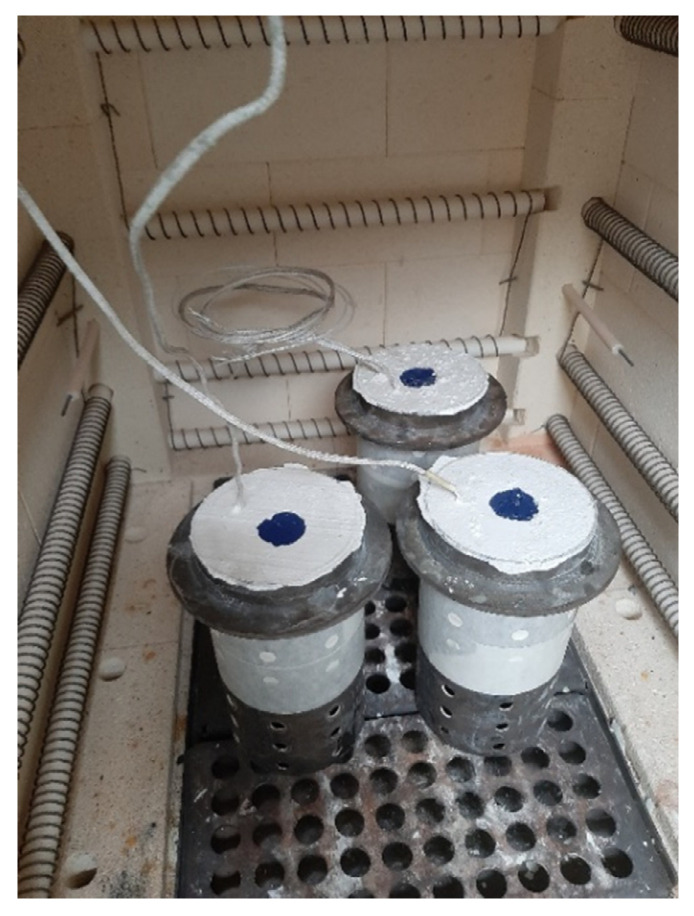
Macroscopic picture of the mold in the furnace before the annealing process.

Thermoelements were connected to the model systems in selected places and located in perforated stainless steel sleeves with the following dimensions: diameter—100 mm; height—200 mm. The sleeves were filled with plastic gypsum mass by Ransom & Randolps, which was prepared from water and gypsum powder in a proportion of 1:2.5, where, after mechanical mixing of components, the mass was degassed in a vacuum chamber [44]. After being tied and dried, the molds prepared in this way were placed in the furnace (Figure 6), where, in the first cycle, the gating system was melted, and, in the next cycle, the molds were annealed in an incremental cycle until the temperature was 720 °C. During this time, the paraffin in the capillaries of the reinforcement was completely gasified, and the mold was completely hardened. The furnace temperature was then lowered to 600 °C where the molds awaited pouring.

**Figure 7:**
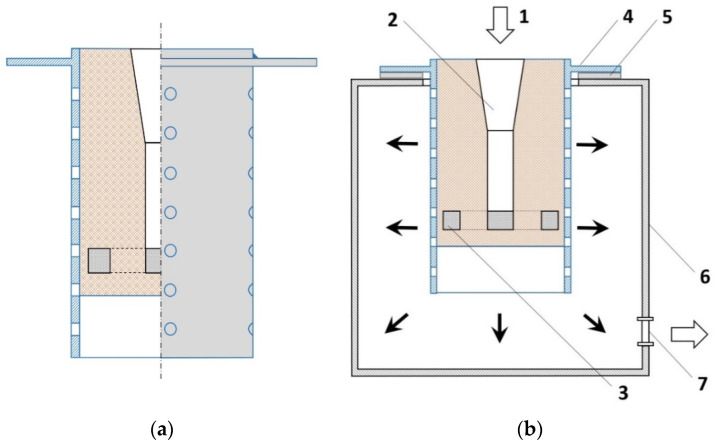
Mold diagram: (**a**) the process of pouring the mold, (**b**) 1—resultant pressure acting on the metal column, 2—main filler, 3—preform, 4—mold ring, 5—gasket, 6—vacuum chamber, 7—suction pipe valve, and arrows—pressure direction.

For infiltration tests, the method of vacuum casting was selected, where infiltration occurs as a result of the difference in pressure in the chamber, as shown in Figure 7. Due to the mold design, which has vent holes drilled in the sleeves and the packing ring, after starting the vacuum pump, the pressure in the chamber is lower than the outside pressure. The resultant sum of the spiral infiltration is metallostatic pressure, resultant atmospheric pressure acting on the metal column, and capillary pressure. A standard, peri-eutectic AlSi11 aluminum alloy with the composition given in Table 2 was selected for pouring.

Eight molds were prepared for saturation tests under constant pressure conditions but with variable temperature parameters. Each time, the procedure was to be conducted as follows:

- Placing the mold on the stand,
- Connecting the measuring elements to the recorder,
- Starting the vacuum pump,
- Pouring liquid alloy into the mold,
- Turning off the pump, after the alloy solidifies in the gating system, and
- Knocking the casting out of the mold.

The first stage of the tests involved determining the temperatures for pouring the mold with the liquid metal and the mold itself at which the spiral will not fill completely. The molds were successively placed on the stand and poured with the alloy. The intended effect was obtained at the alloy pouring temperature of 585 °C and the mold temperature of 580 °C. After determining the expected parameters, subsequent tests were carried out under constant conditions of pressure and alloy temperature, but with successive lowering of the mold temperature. Table 3 and Table 4 present the parameters of ceramic sinter infiltration. The infiltration column in Table 3 presents the values in millimeters. The data presented in Table 3 refer to the degree of saturation of the reinforcement structure with the liquid aluminum alloy in the entire cross-sectional area of the helix and were measured as the distance from the main gating system to the saturation point. These indicate the distance from the front of the spiral and constitute the area (level) of infiltration of the Al_2_O_3_ sinter reinforcement with the matrix metal (AlSi11).

**Figure 8:**
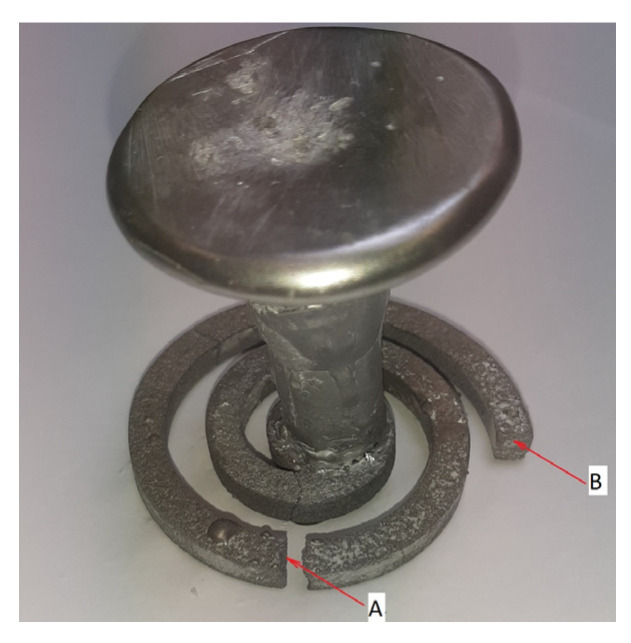
The casting after being knocked out of the mold and cleaned with the sample collection areas marked to assess the degree of filling—exemplary locations for obtaining samples: ½ of spiral length (A); casting boundary area (B).

The castings were knocked out of the molds (Figure 8), the unfilled remains of the reinforcement were removed, and the samples were prepared for metallographic examination in the selected infiltration boundary areas.

## 4. Results

### 4.1. Assessment of the Infiltration Level of the Microstructure with the Use of an Optical Microscope

**Figure 9:**
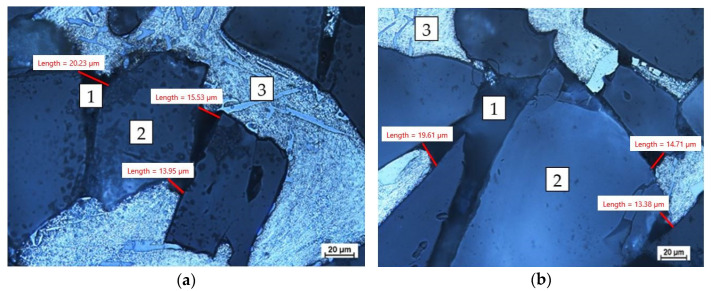
Microscopic picture of the sample from the front of the spiral (1—unfilled capillary, 2—reinforcement, and 3—metal matrix): (**a**) ½ of the spiral length (Figure 8, the area of sample intake marked with the letter A); (**b**) its boundary length (Figure 8, the area of sample intake marked with the letter B), which represents the infiltration boundary area.

First, samples were taken for metallographic evaluation from test no. 3, where the reinforcement was completely infiltrated along its entire length (according to Table 3). The samples were taken in the area in front of the spiral at ½ of the spiral length (Figure 8, the area of sample intake marked with the letter A) and its boundary length (Figure 9, the area of sample intake marked with the letter B). Figure 9 shows the micrographs in selected areas.

The photomicrographs showed areas unfilled by the matrix. Due to the irregular diameter of the capillaries, it can be assumed that the capillaries with a diameter of less than 20 µm were not filled. To verify the correctness of the performed process, estimates were made, determining the minimum diameter of the capillary that will be infiltrated with the liquid matrix. To determine the capillary pressure, P_k,_ the value of the surface tension of the alloy σ = 840 mN/m and the extreme contact angle of the ceramic by the metal θ = 136° were adopted [45,46].

Table 5 presents the estimated pressure values for the adopted capillary diameters. The value of the infiltration pressure, P_n,_ is the sum of the metallostatic pressure and the air pressure outside the chamber minus the pressure in the chamber. The resultant pressure, ΔP, which is the sum of the capillary pressure, P_k,_ and the infiltration pressure, P_n,_ determine the infiltration efficiency for a given capillary diameter. The infiltration condition will be met when ΔP > 0.

**Figure 10:**
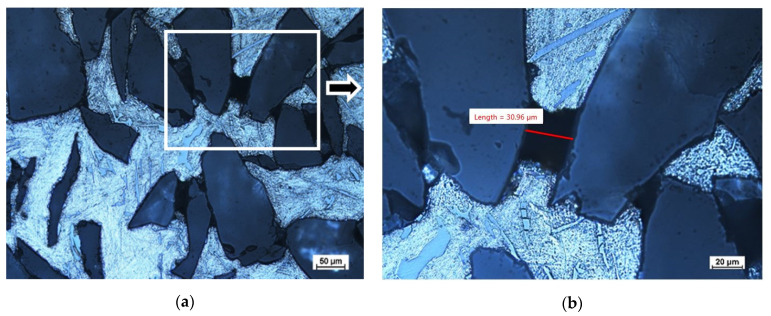
Optical light microscopic images. Evidence for anomalies in the imaging technique using optical microscopy. The unfilled area of Al_2_O_3_ reinforcement with AlSi11 matrix metal of sample no. 3 (designation in accordance with Table 3) at different magnifications: marked area of unfilled capillary measurement (**a**); the value of the unfilled capillary measurement (**b**).

The calculations show that the minimum capillary diameter that will be saturated is 25.08 µm; however, the assumptions may be difficult to determine by means of 2D image analysis. A capillary with a diameter of 25.08 µm has a cross-sectional area of 494 µm^2^ and, in the 2D image, we can only measure distances from one point to another. Figure 10 shows the measurement of an unfilled capillary, where the value of one dimension is 30.96 µm, but, while analyzing the image, we cannot determine (through using an optical microscope) the second dimension, which would allow to calculate the actual cross-section. The problem is the preparation of the sample for analysis, because, by performing the scan, we change the geometry of the prospective measurements. An additional problem is the difficulty of measuring the surface properties of the discussed systems, such as the surface tension and extreme contact angle, as well as deviations from their average values for selected metals given by various researchers [45,47,48].

**Figure 11:**
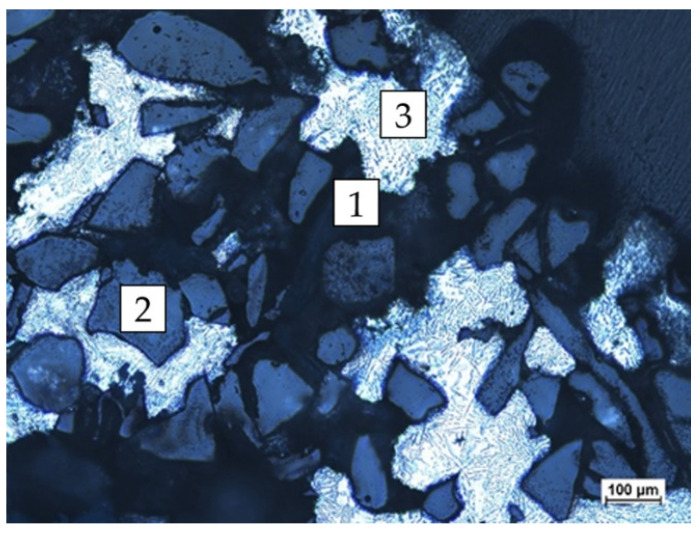
Optical light microscopic images of sample no. 8 documenting the level of saturation. The area of Al_2_O_3_ reinforcement (1—unfilled capillary, 2—reinforcement, and 3—metal matrix).

In the next stage of the research, the limit temperature of the matrix was determined, which would enable infiltration of the reinforcement. Figure 11 shows the saturation boundary area in sample 8 (Table 3), and the length in the spiral where the flow of the matrix stopped is specified in millimeters—154 mm. The distance from the spiral front (in millimeters) is the area of infiltration of the Al_2_O_3_ sinter reinforcement with the matrix metal (AlSi11).

### 4.2. Energy Balance for the Production Process of Castings Reinforced with Al_2_O_3_ Sinter with an AlSi11 Matrix

The energy balance in this study was conducted using empirical data and the forecasted temperature distribution in the mold obtained as a result of numerical simulations. To obtain data for the calculations, validation tests of the thermal model were carried out in the simulation code, the purpose of which was to identify the thermophysical parameters of the casting–mold system and the boundary conditions. The validation tests were carried out with the use of casting experiments in which the temperatures were recorded in selected areas of the casting, reinforcement, and mold. In the conducted validation studies, the thermophysical parameters of the gypsum mold determined in the previous studies were used [49].

The coefficients determined as a result of validation tests enabled us to define the certain conditions and to perform the simulation of the casting process for the saturation parameters listed in Table 3. The simulation tests were carried out according to the following scheme: import of the 3D model and volume mesh generating; implementation of the boundary, initial, material conditions; simulation of the casting process. The simulation tests were carried out with the use of the following substitute thermophysical parameters of the aluminum alloy in the temperature range of 500–600 °C, with the heat conductivity 161–100 [W/(m·K)], specific heat 1056–1000 [J/(kg·K)], density 2556–2356 [kg/m^3^], liquidus temperature T_liq_ = 579.3 °C, and solidus temperature T_sol_ = 573.9 °C. The substitute thermophysical parameters of gypsum mold were assumed as constant values in temperature range of 350–600 °C: heat conductivity 0.12 [W/(m·K)] and volumetric heat capacity 1.44 × 10^6^ [J/(m^3^·K)].

**Figure 12:**
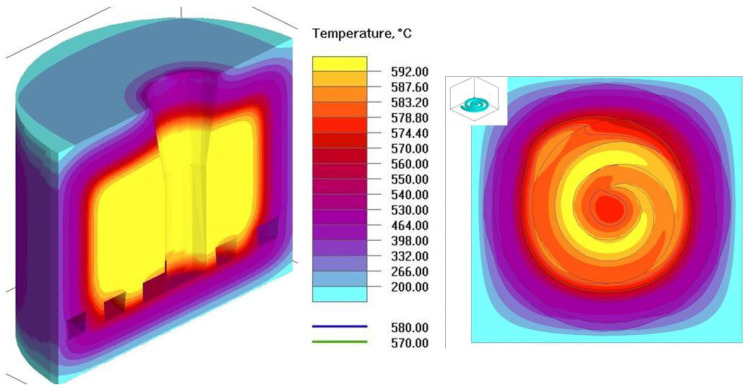
Simulated temperature distribution in the mold and reinforcement for casting no. 8.

The substitute thermophysical parameters of reinforcement were assumed as constant values in the temperature range of 450–600 °C: heat conductivity 1.6 [W/(m·K)] and volumetric heat capacity 0.34 × 10^6^ [J/(m^3^·K)]. The initial temperature of the alloy was 589 °C, and the filling process was started at 425 s of the simulation. The temperature distribution in the mold was simulated for different times after removing the mold from the furnace (initial temperature of the mold and reinforcement—600 °C). As a result of the process of transferring heat by the mold to the environment, the temperature of the mold decreased. Due to the fact that the saturation process started 425 s after removing the mold from the furnace, the temperature in the external parts of the mold dropped below 200 °C. As a result of the simulations, information about the temperature distribution in the mold at the end of the saturation process was obtained. Based on the data from the experiment, the place where the saturation process ended was identified (for casting no. 8 it is 154 mm of the spiral length) and the temperature of the reinforcement and liquid alloy was read in the simulation program at this point. The values of these identified temperatures were used to carry out the energy balance. The exemplary temperature distribution in the mold on the horizontal cross-section of the mold for casting no. 8 from Table 3 is presented in Figure 12.

The energy balance showed that the limit temperature of the feeding was Tk = 580.7 °C (for reinforcement temperature 575 °C). Therefore, the assumption presented in the introduction of the work that the temperature of the metal, after equaling the temperature of the composite components, must be higher than the liquidus temperature (infiltration) has to be met.

## 5. Conclusions

Based on the research conducted, the following conclusions can be made:

1. The use of rapid prototyping techniques for the production of reinforcement allowed for a significant reduction in the time and cost of the research. The reinforcement produced in this way makes it possible to obtain composite castings of a given shape, which is very important from the point of view of the use of castings. The analysis of the impact of polystyrene on ceramics, aluminum matrix requires further research, which exceeded the scope of this work.
2. The tests show that for the conditions presented in Table 3, it is possible to saturate a capillary with a diameter exceeding 25 microns.
3. It is possible by experimental means to determine the basic parameters of infiltration for composite materials. However, the determination of the infiltration level using optical microscopy is poorly precise and requires the use of additional tests, e.g., computer microtomography, which will be the next stage of our work.
4. The experimental results may be helpful for the numerical simulation of the mold-filling process in composite castings with reinforcement infiltration with a liquid silumin matrix.
5. The energy balance shows that the limit temperature of the feeding was Tk = 580.7 °C, and hence this is the temperature above the liquidus temperature (T_liq_ = 579.3 °C) for the tested aluminum alloy.

In the next stage of the research, the authors will assess the porosity of composite shaped castings and characterize selected functional properties.

## Figures and Tables

**Table 1:** Selected physical properties of the produced sinter [43].

Material	Bonding Agent	Grain Sizes of Sintered Particles (μm)	Average Porosity of the Preform (%)	Theoretical Mean Size of Pores (μm)
powder Al_2_O_3_	Water glass	100	46.6	64.6

**Table 2:** Chemical composition of the AlSi11 alloy [%].

Si	Fe	Cu	Mn	Mg	Zn	Ti	Al
11.4	0.14	0.01	0.05	0.4	0.02	0.11	Rest

**Table 3:** Mold and alloy temperature.

No.	Alloy Temp. [°C]	Reinforcement Temp. [°C]	Mold Temp. [°C]	Infiltration [mm]
1	680	600	600	350 (max)
2	640	600	600	350 (max)
3	600	599	590	350 (max)
4	590	599	590	350 (max)
5	585	597	580	163
6	591	582	510	178
7	589	587	546	164
8	589	585	514	154

**Table 4:** The most important parameters of the pouring process.

Parameter	Value
External pressure	0.98 atm
Pressure in chamber	0.05 atm
Infiltration time	15 min

**Table 5:** Resulting pressure for different capillary dimensions.

Capillary Diameter d [µm]	Pressure Value, Pa		
	Capillary Pressure (-) P_k_	Infiltration Pressure (+) P_n_	Resultant Pressure ΔP = P_k_ + P_n_
10	241,698	96,380	−145,318
15	161,132	96,380	−64,752
20	120,849	96,380	−24,469
25	96,679	96,380	−299
25.08	96,370	96,380	10
30	80,566	96,380	15,814
35	69,056	96,380	27,324

## Data Availability

Data is contained within the article.
